# Amla Enhances Mitochondrial Spare Respiratory Capacity by Increasing Mitochondrial Biogenesis and Antioxidant Systems in a Murine Skeletal Muscle Cell Line

**DOI:** 10.1155/2016/1735841

**Published:** 2016-06-02

**Authors:** Hirotaka Yamamoto, Katsutaro Morino, Lemecha Mengistu, Taishi Ishibashi, Kohei Kiriyama, Takao Ikami, Hiroshi Maegawa

**Affiliations:** ^1^Institute for Health Science, MIKI Corporation, 3-12-4, Naruohama, Nishinomiya, Hyogo 663-8142, Japan; ^2^Department of Medicine, Division of Endocrinology and Metabolism, Shiga University of Medical Science, Tsukinowa, Seta, Otsu, Shiga 520-2192, Japan

## Abstract

Amla is one of the most important plants in Indian traditional medicine and has been shown to improve various age-related disorders while decreasing oxidative stress. Mitochondrial dysfunction is a proposed cause of aging through elevated oxidative stress. In this study, we investigated the effects of Amla on mitochondrial function in C2C12 myotubes, a murine skeletal muscle cell model with abundant mitochondria. Based on cell flux analysis, treatment with an extract of Amla fruit enhanced mitochondrial spare respiratory capacity, which enables cells to overcome various stresses. To further explore the mechanisms underlying these effects on mitochondrial function, we analyzed mitochondrial biogenesis and antioxidant systems, both proposed regulators of mitochondrial spare respiratory capacity. We found that Amla treatment stimulated both systems accompanied by AMPK and Nrf2 activation. Furthermore, we found that Amla treatment exhibited cytoprotective effects and lowered reactive oxygen species (ROS) levels in cells subjected to t-BHP-induced oxidative stress. These effects were accompanied by increased oxygen consumption, suggesting that Amla protected cells against oxidative stress by using enhanced spare respiratory capacity to produce more energy. Thus we identified protective effects of Amla, involving activation of mitochondrial function, which potentially explain its various effects on age-related disorders.

## 1. Introduction

Amla is one of the most important botanical materials in Indian traditional medicine, “Ayurveda.” It has been used for many diseases including diabetes, osteoporosis, liver dysfunction, and anemia, not only in India but also in other countries [1, 2]. Recently, the chemical composition of Amla was analyzed, showing relatively high levels of phenolic compounds [2]. A recent clinical study showed that Amla extract improved endothelial function in patients with type 2 diabetes mellitus [3]. Furthermore, its antioxidant [4], hepatoprotective [5], nephroprotective [6], hypolipidemic [7, 8], cardioprotective [9, 10], and antidiabetic effects [11] were demonstrated in* in vivo* animal models. One potential mechanism for these effects was reduction of oxidative stress, based on observations of decreased oxidative stress with Amla treatment. However, the detailed mechanisms have not yet been fully identified.

Accumulating evidence suggested a central role for mitochondria in aging through production of both energy and reactive oxygen species (ROS). Mitochondria can produce substantial energy through aerobic metabolism, though they also produce ROS as unwanted byproducts, causing serious damage to various cellular components. Because mitochondria are the major ROS producing organelle, they are also vulnerable to injury by ROS. Damaged mitochondria produce more ROS, leading to further mitochondrial damage [12]. These defective mitochondria exhibit impaired energy production and increased oxidative stress, both negatively impacting cellular function. Numerous reports implicated mitochondrial dysfunction in many pathologies or disorders related to the aging process [13, 14]. Mitochondrial spare respiratory capacity is regarded as an important aspect of mitochondrial function and is defined as the difference between basal ATP production and its maximal activity. When cells are subjected to stress, energy demand increases, with more ATP required to maintain cellular functions. A cell with a larger spare respiratory capacity can produce more ATP and overcome more stress, including oxidative stress [15]. Therefore, we hypothesized that Amla could improve mitochondrial function, especially spare respiratory capacity, and exert positively effects on various disorders related to oxidative stress.

To explore the effects of Amla on mitochondrial function, we used a skeletal muscle cell known to contain abundant mitochondria. Using this model, we investigated the molecular mechanisms of Amla's beneficial effects.

## 2. Materials and Methods

### 2.1. Plant Materials

A commercial product of Amla fruit juice extract (SunAmla) was obtained from Taiyo Kagaku Co., Ltd. (Mie, Japan). It is a dried powder of water extract from fresh Amla fruit and was previously shown to contain ~30% polyphenols and 2% vitamin C [6]. To confirm the equivalence of Amla used in this study, total polyphenol was analyzed by a colorimetric method using gallic acid as a standard [6], and vitamin C content was analyzed by high-performance liquid chromatography (HPLC) as previously reported [16]. Glucose and fructose in Amla were analyzed by HPLC as previously reported [17]. To prepare Amla extract stock solution, the extract was dissolved in distilled water at a concentration of 200 mg/mL and then sterilized using a polyvinylidene difluoride membrane filter (Merck Millipore, Darmstadt, Germany).

### 2.2. Reagents

Oligomycin, carbonyl cyanide-p-trifluoromethoxyphenylhydrazone (FCCP), rotenone, and antimycin A were obtained from Seahorse Bioscience (North Billerica, MA, USA) in XF Cell Mito Stress Test kit (#103015-100). Water soluble tetrazolium salts for the MTT assay were from Kishida Chemical Co., Ltd. (Osaka, Japan). 2′,7′-Dichlorofluorescein diacetate (D6883) and* tert*-butyl hydroperoxide (t-BHP; 458139) were obtained from Sigma-Aldrich (St. Louis, MO, USA).

### 2.3. Cell Culture

C2C12 myoblasts were obtained from the American Type Culture Collection (ATCC; Manassas, VA, USA) and grown in Dulbecco's modified Eagle's medium (DMEM) supplemented with 10% (v/v) fetal bovine serum (FBS), 100 units/mL penicillin, and 100 mg/mL streptomycin in a humidified atmosphere of 95% air and 5% CO_2_ at 37°C. At confluence, myoblasts were induced to differentiate in DMEM with 2% FBS, 100 units/mL penicillin, and 100 mg/mL streptomycin. Differentiation medium was replaced every 48 h. Human embryonic kidney 293 (HEK293) cells were obtained from the Japanese Collection of Research Bioresources cell bank (Osaka, Japan) and grown in Eagle's minimum essential medium with 10% (v/v) FBS, 100 units/mL penicillin, and 100 mg/mL streptomycin in a humidified atmosphere of 95% air and 5% CO_2_ at 37°C. For Amla treatment, Amla stock solution was diluted with medium to final concentrations of 100 *μ*g/mL and 200 *μ*g/mL.

### 2.4. Measurement of Mitochondrial Function

**Figure 1:**
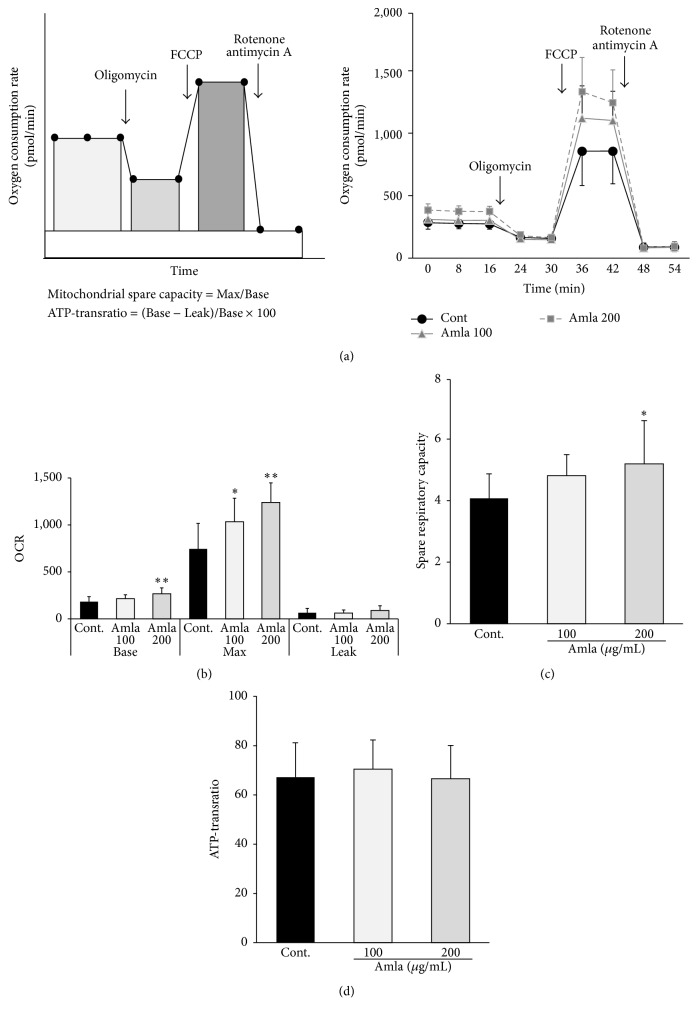
Amla treatment stimulated mitochondrial bioenergetic function. C2C12 myotubes were pretreated with two doses of Amla (100 *μ*g/mL or 200 *μ*g/mL) for 48 h and subjected to mitochondrial function analysis. ((a), left panel) Schematic figure analyzing mitochondrial function using an extracellular flux analyzer. After measuring basal OCR, oligomycin, FCCP, and rotenone/antimycin A were sequentially injected to measure OCR from proton leak, maximal respiratory capacity, and nonmitochondrial respiration, respectively. OCR from nonmitochondrial respiration was subtracted from OCR at each stage to calculate the net OCR for basal (Base), proton leak (Leak), and maximal respiratory capacity (Max) values. Mitochondrial spare respiratory capacity and ATP-transratio were calculated by the formula shown. ((a), right panel) OCR measurements over time (*n* = 10 or 11). (b) Basal, Max, and Leak OCRs represented as average values from multiple measurements. (c) Mitochondrial spare respiratory capacity was calculated from Max and Basal OCRs. (d) ATP-transratio was calculated from Basal and Leak OCRs. ^*∗*^ *p* < 0.05; ^*∗∗*^ *p* < 0.01 as compared with control (*n* = 10 or 11).

Mitochondrial function in C2C12 myotubes was analyzed using the XFe24 flux analyzer with XF Cell Mito Stress Test kit according to manufacturer instructions (Seahorse Bioscience). Briefly, differentiated C2C12 myotubes were prepared on Seahorse 24-well plates and treated with or without Amla (100 *μ*g/mL or 200 *μ*g/mL) for 48 h. The culture medium was changed at least 40 min prior to the assay to unbuffered DMEM supplemented with 5 mM glucose. The oxygen consumption ratio (OCR, pmol/min) was monitored in real time, with sequential treatments with oligomycin (ATP synthase inhibitor), FCCP (mitochondrial uncoupler), and rotenone/antimycin A (respiration inhibitor) to evaluate OCR from proton leak, maximum respiration capacity, and nonmitochondrial respiration, respectively. OCR was measured multiple times at 8 min intervals at each stage, and average values were determined. Nonmitochondrial respiration was subtracted from OCR at each stage to calculate the net OCR for Basal, Leak, and Max values. Mitochondrial respiratory spare capacity and ATP-transratio were calculated by the formula shown in Figure 1(a), left panel.

### 2.5. Nucleic Acid and Protein Isolation

Total DNA was isolated using a Qiamp DNA mini kit (Qiagen, Mississauga, ON, Canada) according to manufacturer instructions. RNA and total protein were isolated using a PARIS kit (AM1921; Thermo Fisher Scientific, Waltham, MA, USA). Nuclear lysates were isolated using a nuclear/cytosol-fractionation kit (K266; Biovision, Inc., Milpitas, CA, USA) according to manufacturer protocol. Isolated protein samples were denatured by boiling in sodium dodecyl sulfate (SDS) sample buffer.

### 2.6. Western Blot

Proteins were separated by SDS-polyacrylamide gel electrophoresis and transferred onto polyvinylidene fluoride membranes. Immunoblotting was performed with primary antibodies against phosphorylated AMPK*α* (#2531; Cell Signaling Technology, Danvers, MA, USA), AMPK*α* (#2603; Cell Signaling Technology), Nrf2 (SC-722; Santa Cruz Biotechnology, Dallas, TX, USA), and YY-1 (ab109237; Abcam, Tokyo, Japan), with horseradish peroxidase-conjugated secondary antibodies (GE Healthcare Japan, Tokyo, Japan). YY-1 was used as a nuclear-loading control. The intensity of protein bands was visualized using a chemiluminescence detection reagent (PerkinElmer, Inc., Waltham, MA, USA) and a WSE-6100 luminograph system (ATTO, Tokyo, Japan).

### 2.7. Quantitative PCR for Mitochondrial DNA (mtDNA) Content

mtDNA content was analyzed as previously described [18]. DNA primers were designed to detect cytochrome oxidase 2 (COX2) and uncoupling protein 2 (UCP2) for mtDNA and nuclear DNA, respectively (COX2-F: 5′-TTTTCAGGCTTCACCCTAGATGA-3′ COX2-R: 5′-GAAGAATGTTATGTTATGTTTACTCCTA-3′ UCP2-F: 5′-GCGACCAGCCCATTGTAGA-3′ UCP2-R: 5′-GCGTTCTGGGTACCATCCTAAC-3′). The ratio of COX2 to UCP2 within each sample was used to calculate mtDNA content.

### 2.8. mRNA Quantification

Complementary DNA was prepared using the PrimeScript2 1st strand cDNA synthesis kit (Takara, Otsu, Japan). RT-qPCR was performed in a StepOnePlus Real-Time PCR system (Thermo Fisher Scientific) using Fast SYBR Green Master Mix (Thermo Fisher Scientific) and primer pair sets described in Table 1. The 18S rRNA was used as a housekeeping gene and served as an endogenous control.

### 2.9. Antioxidant Response Element (ARE) Luciferase Assay

The luciferase reporter plasmid (pGL4.26 luc2/minP/Hygro, E8441) and internal control plasmid (pRL-CMV, E2271) were from Promega (Madison, WI, USA). The DNA sequence for the ARE was obtained from the National Center for Biotechnology Information (GenBank: JQ858521.1), and a synthetic ARE polynucleotide was inserted into the* Hin*dΙΙΙ site in multicloning site of pGL4.26 using the In-Fusion HD cloning kit (Takara). The ARE-loaded reporter and internal control vectors were cotransfected into HEK293 cells using lipofectamine-transfection reagent (Thermo Fisher Scientific). After a 24 h incubation, cells were treated with Amla (200 *μ*g/mL) for another 48 h, and luciferase activity was measured using the Dual-Luciferase Reporter Assay System (Promega).

### 2.10. MTT Measurements of Cell Viability under Oxidative Stress

C2C12 cells were seeded on 96-well plates and differentiated to myotubes as previously described in Section 2.3. To evaluate the cytoprotective effects of Amla on t-BHP-induced oxidative stress, cells were pretreated with or without Amla (200 *μ*g/mL) for 48 h, then with t-BHP (250 *μ*M or 500 *μ*M) for 6 h. Cell viability was determined using a cell-viability kit based on MTT reduction. Briefly, after treatment with t-BHP, cells were incubated with MTT assay reagents in culture medium for 20 min, and absorbance at 495 nm was measured using an ARVO SX 1420 multilabel counter (PerkinElmer, Inc., Waltham, MA, USA). Cell viability was expressed as the percentage of values obtained with control cells not treated with t-BHP.

### 2.11. ROS Measurement

Intracellular ROS levels were analyzed using the fluorescent probe 2′,7′-dichlorofluorescein diacetate. Differentiated C2C12 myotubes in 96-well culture plates were pretreated with Amla (200 *μ*g/mL) for 48 h. Myotubes were incubated with 10 *μ*M 2′,7′-dichlorofluorescein diacetate for 30 min following t-BHP treatment (0 *μ*M, 250 *μ*M, or 500 *μ*M) for 2 h. The fluorescence in cells, which was increased by ROS, was analyzed at 485 nm/535 nm (excitation/emission) in an ARVO SX 1420 multilabel counter.

### 2.12. Oxygen Consumption Analysis in Response to Oxidative Stress

Amla (200 *μ*g/mL) treated or untreated C2C12 myotubes in 24-well plates (Seahorse Bioscience) were prepared as described in Section 2.4. Basal OCR was measured three times at 8 min intervals prior to treatment. After administration of t-BHP (0 *μ*M, 250 *μ*M, or 500 *μ*M), OCR was then measured at 8 min intervals for 160 min. Data are expressed as relative-OCR values normalized to basal OCR values.

### 2.13. Statistical Analysis

Results are means ± standard deviation. Significance was assessed using Student's *t*-test and one-way analysis of variance. Differences among groups were determined using Tukey's multiple range test. A *p* < 0.05 was considered significant.

## 3. Results

### 3.1. Amla Extract Components

The total polyphenol and vitamin C contents of Amla extract were 23.9% and 1.32%, respectively (Table 2), and the extract contained low levels of glucose and fructose (3.5% and 4.3%, resp.). Because Amla extract was applied to cells at a concentration of 200 *μ*g/mL, extract-derived sugars (~0.8 mg/dL each) were regarded as negligible.

### 3.2. Amla Treatment Enhanced Mitochondrial Spare Respiratory Capacity

To evaluate the effects of Amla treatment on mitochondrial function, we analyzed the OCR in C2C12 myotubes with or without Amla pretreatment (100 *μ*g/mL or 200 *μ*g/mL). Nonmitochondrial respiration (Figure 1(a), right panel) and Leak (Figure 1(a), right panel, and Figure 1(b)) were unchanged by Amla treatment; however, Base and Max OCRs increased in a dose-dependent manner following Amla treatment (Figure 1(a), right panel, and Figure 1(b)). Mitochondrial spare respiratory capacity also increased in a dose-dependent manner following Amla treatment, though the effects were not significant at lower Amla doses (Figure 1(c)). The ATP-transratio was unchanged by Amla treatment (Figure 1(d)). In order to clarify the mechanisms associated with Amla treatment, we conducted further experiments using the higher dosage (200 *μ*g/mL).

### 3.3. Amla Treatment Stimulated Mitochondrial Biogenesis by AMPK Activation

**Figure 2:**
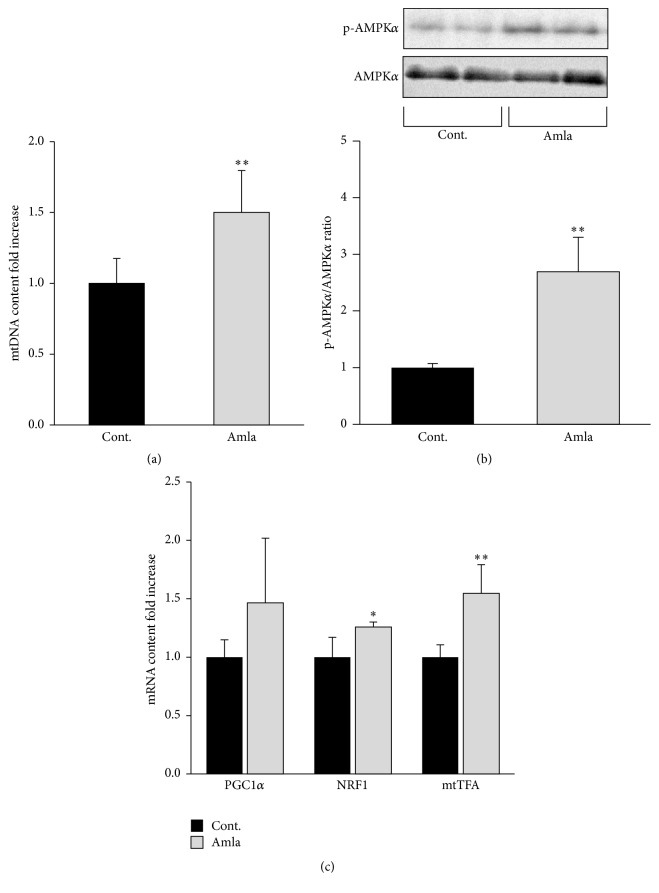
Amla treatment stimulated mitochondrial biogenesis by AMPK activation. C2C12 myotubes were incubated with Amla (200 *μ*g/mL) for 48 h. Cell lysates were prepared for western blotting, RT-qPCR, and mtDNA analysis. (a) Relative mtDNA content was determined by qPCR using specific primer sets for the mitochondrial and nuclear genome. ^*∗∗*^ *p* < 0.01; *n* = 8. (b) Phosphorylated AMPK*α* to total AMPK*α* ratios were determined by western blot. ^*∗∗*^ *p* < 0.01; *n* = 6. (c) Relative contents of PGC1*α*, NRF1, and mtTFA mRNAs were determined by RT-qPCR. ^*∗*^ *p* < 0.05 and ^*∗∗*^ *p* < 0.01; *n* = 5. 18S rRNA was used as an internal control for RT-qPCR.

To elucidate how Amla treatment enhanced OCR and mitochondrial spare respiratory capacity, we evaluated its effects on mitochondrial biogenesis in C2C12 myotubes. mtDNA copy number increased 1.5-fold following Amla treatment (Figure 2(a)). To evaluate the molecular effects associated with Amla treatment, we analyzed activation of the AMPK*α*/PGC1*α*/NRF1/mtTFA pathway, a key regulatory pathway involved in mitochondrial biogenesis. In cells treated with Amla, AMPK*α*-phosphorylation levels increased dramatically (Figure 2(b)), NRF1 and mtTFA mRNA expression increased significantly, and PGC1*α* mRNA exhibited trends indicating increased expression levels (Figure 2(c)).

### 3.4. Amla Treatment Stimulated Antioxidant Systems

**Figure 3:**
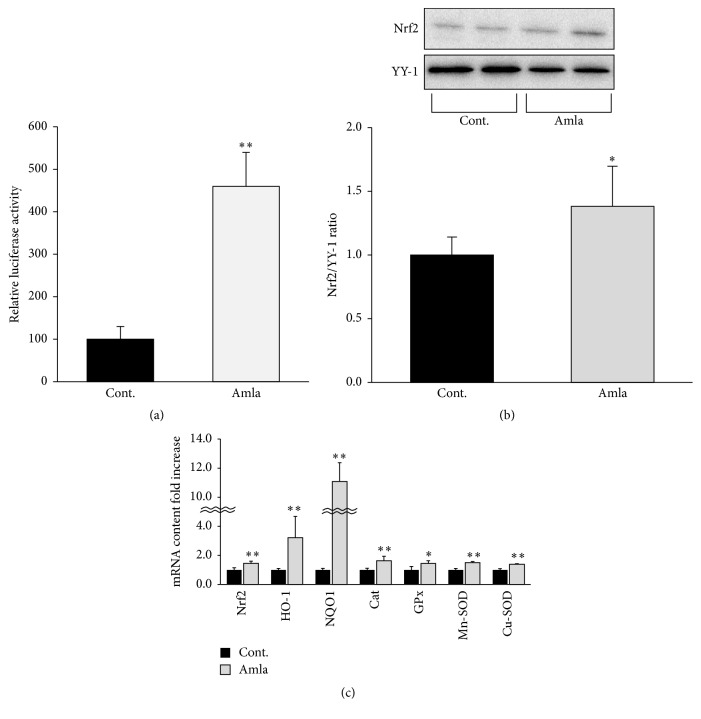
Amla treatment stimulated antioxidant systems by Nrf2 activation. (a) Activation of Nrf2 was analyzed using an ARE luciferase assay. Data are expressed as relative activities (reporter luciferase activity/control luciferase activity) as compared with data from control cells. ^*∗∗*^ *p* < 0.01; *n* = 5. (b and c) C2C12 myotubes were incubated with Amla (200 *μ*g/mL) for 48 h. (b) Nuclear lysates were analyzed by western blot, and YY-1 was used an internal control for nuclear protein. ^*∗*^ *p* < 0.05; *n* = 6. (c) Relative levels of mRNA for antioxidant system related genes were analyzed by RT-qPCR. ^*∗*^ *p* < 0.05; ^*∗∗*^ *p* < 0.01; *n* = 5. 18S rRNA was used as an internal control for RT-qPCR.

In order to maintain mitochondrial function, ROS reduction and mitochondrial biogenesis are important. Therefore, we assessed the effects of Amla treatment on antioxidant systems in C2C12 myotubes. The activity of Nrf2, a key transcriptional factor involved in cellular antioxidant systems, was analyzed by luciferase reporter assay using a vector containing an ARE. As shown in Figure 3(a), Amla treatment dramatically increased ARE-driven luciferase activity. To further evaluate Nrf2 activation, we analyzed translocation of Nrf2 and observed that Amla treatment significantly increased Nrf2 translocation to the nucleus in C2C12 myotubes (Figure 3(b)). Furthermore, we found that several antioxidant enzymes that are Nrf2 target genes, such as HO-1, NQO-1, Cat, Mn-SOD, and Cu-SOD, were significantly upregulated at the transcriptional level following Amla treatment (Figure 3(c)).

### 3.5. Amla Treatment Exhibited Cytoprotective Effects on Cells Subjected to Oxidative Stress

**Figure 4:**
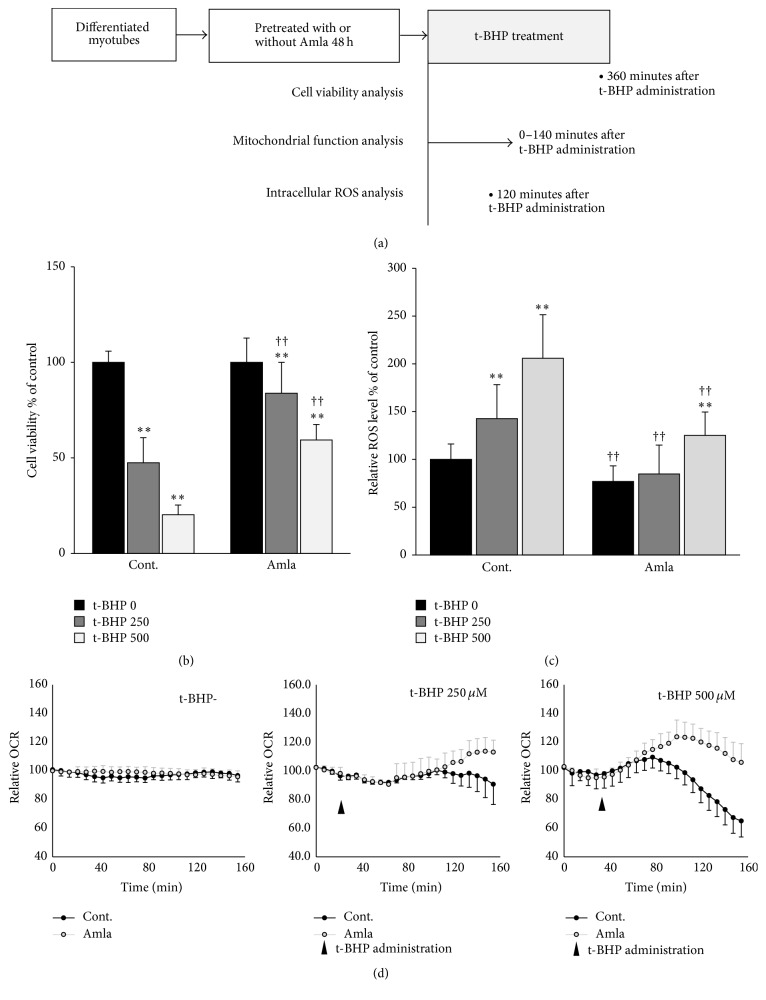
Amla treatment exhibited a cytoprotective effect against oxidative stress and concomitantly increased oxygen consumption. C2C12 myotubes were pretreated with Amla (200 *μ*g/mL) for 48 h and then treated with t-BHP (250 *μ*M  or 500 *μ*M). (a) Schematic showing the time points for three experiments performed to evaluate the cytoprotective effects of Amla treatment. (b) Cell viability was analyzed by MTT assay at 6 h after t-BHP treatment. ^*∗∗*^ *p* < 0.01 versus t-BHP-untreated cells; ^††^ *p* < 0.01 versus Amla-untreated cells treated with each t-BHP concentration; *n* = 20. (c) Relative ROS levels in cells were analyzed at 2 h after t-BHP stimulation. ^*∗∗*^ *p* < 0.01 versus t-BHP-untreated cells; ^††^ *p* < 0.01 versus Amla-untreated cells treated with each t-BHP concentration; *n* = 12. (d) OCR after t-BHP stimulation was analyzed following t-BHP injection after three basal OCR measurements. OCR was measured every 8 min for a total of 160 min. Data are represented as relative-OCR values divided by the basal OCR values measured prior to t-BHP treatment (*n* = 10).

To evaluate the cytoprotective effects of Amla treatment against oxidative stress, we first evaluated cell viability using the MTT assay in cells treated with t-BHP for 6 h to induce oxidative stress (Figure 4(a)). As shown in Figure 4(b), t-BHP treatment (250 *μ*M and 500 *μ*M) induced cytotoxicity in a dose-dependent manner, whereas this was significantly attenuated by pretreatment with Amla for 48 h. Next, to evaluate oxidative stress status, we analyzed ROS levels in C2C12 myotubes treated with t-BHP for 2 h (Figure 4(a)). Without t-BHP treatment, Amla pretreatment significantly reduced ROS levels, whereas t-BHP treatment increased ROS levels in a dose-dependent manner, which was significantly suppressed by pretreatment with Amla for 48 h (Figure 4(c)). We then evaluated oxygen consumption under oxidative stress conditions in C2C12 myotubes with or without Amla pretreatment (Figure 4(a)). In control myotubes, t-BHP at high doses caused a time-dependent increase in oxygen consumption, followed by a gradual decline, exhibiting a maximum OCR of 109.5 ± 7.3% that of basal OCR. Amla pretreatment increased maximum OCR in the presence of t-BHP (113.7 ± 9.7% and 120.6 ± 10.0% that of basal OCR at 250 *μ*M and 500 *μ*M t-BHP, resp.). OCRs were unchanged following Amla pretreatment in the absence of t-BHP treatment (Figure 4(d)).

## 4. Discussion

In this study, we made three observations relevant to the beneficial effects of Amla treatment in murine skeletal muscle cells. First, Amla treatment stimulated mitochondrial function, specifically increasing mitochondrial spare respiratory capacity. Second, Amla treatment stimulated mitochondrial biogenesis and antioxidant systems along with activation of the AMPK and Nrf2 pathways. Third, Amla treatment protected cells against oxidative stress accompanied by increased oxygen consumption.

Amla treatment stimulated mitochondrial function by increasing mitochondrial spare respiratory capacity (Figure 1). There have been few studies reporting the ability of food ingredients to enhance mitochondrial spare respiratory capacity. In mouse cortical neuronal cultures, epicatechin and quercetin, both major polyphenols in plant-derived food materials, enhanced mitochondrial spare respiratory capacity and protected cells against oxygen-glucose deprivation stress [19]. Additionally, pharmaceutical approaches, such as administering thyroid hormones, can positively regulate mitochondrial spare respiratory capacity in skeletal muscle [20]. Decreased mitochondrial spare respiratory capacity was also reported in aging [21], and its depletion was implicated in various pathologies in high energy-requiring tissues, such as the heart, brain, and skeletal muscle [22–24]. Our findings suggested that Amla and other food ingredients are potentially novel approaches to improving age-related disorders in skeletal muscle by enhancing mitochondrial spare respiratory capacity. We confirmed that polyphenol rich Amla fractions prepared by adsorption chromatography stimulated mitochondrial respiratory spare capacity (see Supplemental Figures 1(A)–1(C) in Supplementary Material available online at http://dx.doi.org/10.1155/2016/1735841). These results indicated that the polyphenols were the functional components responsible for the observed effects, and given that Amla was reported to contain higher polyphenol content [6], it was expected to be superior to other fruits in mitochondrial maintenance.

Amla treatment stimulated mitochondrial biogenesis and antioxidant systems along with activation of the AMPK*α* and Nrf2 pathways (Figures 2 and 3). Studies showed that both mitochondrial biogenesis and antioxidant systems could regulate mitochondrial spare respiratory capacity. Increased mitochondrial biogenesis and energy production caused by thyroid hormone treatment leads to stimulation of mitochondrial spare respiratory capacity in skeletal muscle [20]. In striatal neurons, decreased mitochondrial density accompanying mutant huntingtin expression indicated reduced mitochondrial spare respiratory capacity [25]. Furthermore, depletion of an antioxidant enzyme impaired mitochondrial spare respiratory capacity in cortical synaptosomes isolated from* SOD2* null mice [26]. These reports were consistent with our findings, and we speculated that the effects of Amla treatment on mitochondrial spare respiratory capacity were related to activation of mitochondrial biogenesis and antioxidant systems. AMPK*α* and Nrf2 activation reportedly play key roles in mitochondrial biogenesis and antioxidant systems, and there are many studies showing that plant-derived polyphenols activate AMPK*α* or Nrf2 [27, 28]. In an animal model, dietary gallic acid protected mice from diet-induced obesity by stimulating the AMPK*α*/Sirt1/PGC1*α* pathway in liver, muscle, and brown adipose tissues [29]. Furthermore, ellagic acid consumption improved oxidant-induced endothelial dysfunction through Nrf2 activation [30]. These reports supported our findings, because the major polyphenols in Amla include gallic and ellagic acid [6]. Therefore, we proposed that gallic acid and ellagic acid were key factors involved in AMPK and Nrf2 activation following Amla treatment in our experiments.

Amla treatment protected cells against oxidative stress accompanied by increased oxygen consumption (Figure 4). Many reports showed that activation of antioxidant systems could increase cell survival in the presence of oxidative stress [31, 32]. In our study, we observed that Amla treatment protected cells against t-BHP-induced cell death, likely by reducing ROS levels through activation of the Nrf2 pathway (Figures 4(b) and 4(c)). Various oxidative stresses led to increased oxygen consumption, suggesting a critical role for mitochondrial spare respiratory capacity in cell survival and maintenance of biological functions under oxidative stress [15, 33]. We observed that t-BHP treatment increased OCR and that this was likely mediated by the spare respiratory capacity against oxidative stress. Additionally, pretreatment with Amla elevated the maximum OCR against t-BHP treatment (Figure 4(d)). Therefore, we speculated that not only activation of antioxidant systems but also increased mitochondrial function contributed to the cytoprotective effects of Amla treatment against oxidative stress.

In addition to being toxic, ROS are involved in various physiological processes as messenger molecules [34]. Signaling by insulin, several growth factors, and transcriptional factors can be mediated by physiological ROS levels [35, 36]. Because of these multiple roles, ROS homeostasis is regulated by numerous systems [37]. In our experiments, the magnitude by which Amla treatment decreased ROS increased with t-BHP concentration (Figure 4(c)). Therefore, our findings suggested that Amla treatment might remove excessive and damaging levels of ROS without neutralizing the ROS required for physiological function.

One limitation of our study was that it only demonstrated the effects of Amla treatment* in vitro*. Component analysis and previous research strongly suggested that polyphenols contributed to the effects of Amla treatment [29, 30] and that Amla contained various kinds of polyphenols, with gallic acid as a major component [6]. In one report, gallic acid was absorbed better in humans as compared to other polyphenols [38], and a previous clinical study investigating the bioavailability of gallic acid from red wine showed that glucuronidated and intact forms of gallic acid were detected in plasma [39]. These findings indicated that gallic acid in Amla may partially account for the biological relevance of our findings. Further experiments will be needed to clarify whether the effects of Amla treatment reported here can be reproduced* in vivo*.

**Figure 5:**
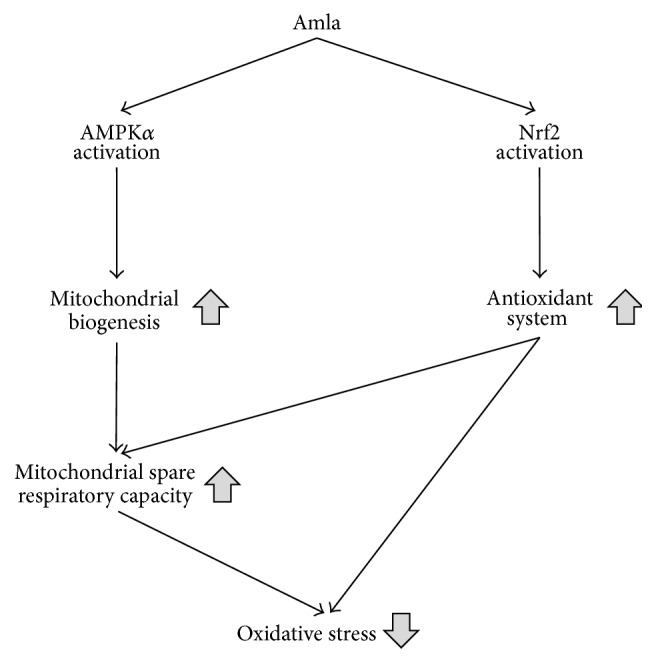
Schematic figure showing the effects of Amla treatment resulting in reduced oxidative stress.

Our results indicated that Amla treatment enhanced mitochondrial spare respiratory capacity, which was supported by its effect on mitochondrial biogenesis and antioxidant systems, through activation of the AMPK*α* and Nrf2 pathways, respectively. Furthermore, we found that Amla treatment decreased ROS levels and increased cell viability in the presence of t-BHP induced oxidative stress. We attributed the cytoprotective effects of Amla treatment not only to activation of antioxidant systems, but also to enhancement of the mitochondrial spare respiratory capacity (Figure 5).

## 5. Conclusions

In conclusion, our study demonstrated that Amla enhanced mitochondrial spare respiratory capacity through activation of mitochondrial biogenesis and antioxidant systems. Furthermore, we showed that these effects of Amla resulted in decreased ROS levels and increased cell viability under oxidative stress conditions. Therefore, our findings suggested novel potential mechanisms for the beneficial effects associated with Amla intake, including decreased oxidative stress.

## Supplementary Material

Amla treatment stimulated mitochondrial bioenergetics function. To explore an active integrant in Amla, Amla extract was separated by reversed-phase chromatography to obtain a polyphenol-rich fraction (a mixture of polyphenols corresponding to what is present in the Amla fruit) and a polyphenol-poor fraction (a mixture of the constituents of Amla extract without polyphenols). We analyzed mitochondrial function and found that the effect of Amla treatment was due to the polyphenol-rich fraction. These results indicated that the polyphenols were the functional components responsible for the observed effects.

## Figures and Tables

**Table 1:** Primer sets used for RT-qPCR.

Gene name	F/R	Sequence (5′ to 3′)
Internal standard		
*18S rRNA*	F	TTCCGATAACGAACGAGACTCT
	R	TGGCTGAACGCCACTTGTC
Mitochondrial biogenesis		
*PGC1α*	F	CCAAACCCACAGAAAACAGG
*PPAR-gamma coactivator 1-alpha *	R	TGGGGTCATTTGGTGACTCT
*NRF1*	F	GAACTGCCAACCACAGTCAC
*Nuclear respiratory factor 1*	R	TCGTCTGGATGGTCATTTCA
*mtTFA*	F	CCGAAGTGTTTTTCCAGCAT
*Mitochondrial transcriptional factor A*	R	GGCTGCAATTTTCCTAACCA
Antioxidant system		
*Nrf2*	F	GGGAGAAAACGACAGAAACC
*Nuclear factor erythroid 2 related factor 2*	R	TGGGAGAGTAAGGCTTTCCA
*HO-1*	F	TGACACCTGAGGTCAAGCAC
*Heme oxygenase-1*	R	TCCTCTGTCAGCATCACCTG
*NQO1*	F	AAACGTCTGGAAACCGTCTG
*Nadph dehydrogenase quinone 1*	R	TTCTGCTCCTCTTGAACTTCC
*Cat*	F	GGACGCTCAGCTTTTCATTC
*Catalase*	R	TTGTCCAGAAGAGCCTGGAT
*GPx*	F	CTCATGACCGACCCCAAGTA
*Glutathione peroxidase*	R	CCCACCAGGAACTTCTCAAA
*Mn-SOD*	F	TCTGTGGGAGTCCAAGGTTC
*Manganese superoxide dismutase*	R	TAAGGCCTGTTGTTCCTTGC
*Cu-SOD*	F	GAGACCTGGGCAATGTGACT
*Copper superoxide dismutase*	R	TCATGGACCACCATTGTACG

**Table 2:** Components of Amla extract.

	Content, %	
	Mean	SD
Total polyphenols	23.9	0.6
Vitamin C	1.32	0.2
Glucose	3.5	0.2
Fructose	4.3	0.4

Values are means of three replicate samples.
