# Supplementary material for: Amla Enhances Mitochondrial Spare Respiratory Capacity by Increasing Mitochondrial Biogenesis and Antioxidant Systems in a Murine Skeletal Muscle Cell Line
Source: Oxid Med Cell Longev. 2016 Jun 2;2016:1735841. doi: 10.1155/2016/1735841 (PMC4909908; doi:10.1155/2016/1735841)
Supplement: Supplementary file 1 — Amla treatment stimulated mitochondrial bioenergetics function. To explore an active integrant in Amla, Amla extract was separated by reversed-phase chromatography to obtain a polyphenol-rich fraction (a mixture of polyphenols corresponding to what is present in the Amla fruit) and a polyphenol-poor fraction (a mixture of the constituents of Amla extract without polyphenols). We analyzed mitochondrial function and found that the effect of Amla treatment was due to the polyphenol-rich fraction. These results indicated that the polyphenols were the functional components responsible for the observed effects. [file 1735841.f1.pdf]

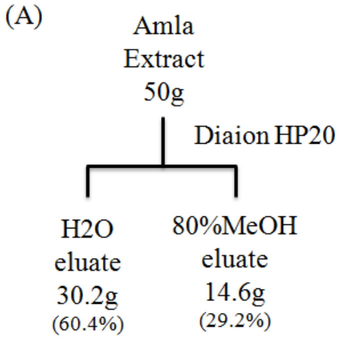

(B)

	Content %	
	H2O eluate	80%MeOH eluate
polyphenol	5.0	54.7
vitamin C	nd	3.1
Glucose	5.6	nd
Fructose	6.6	nd

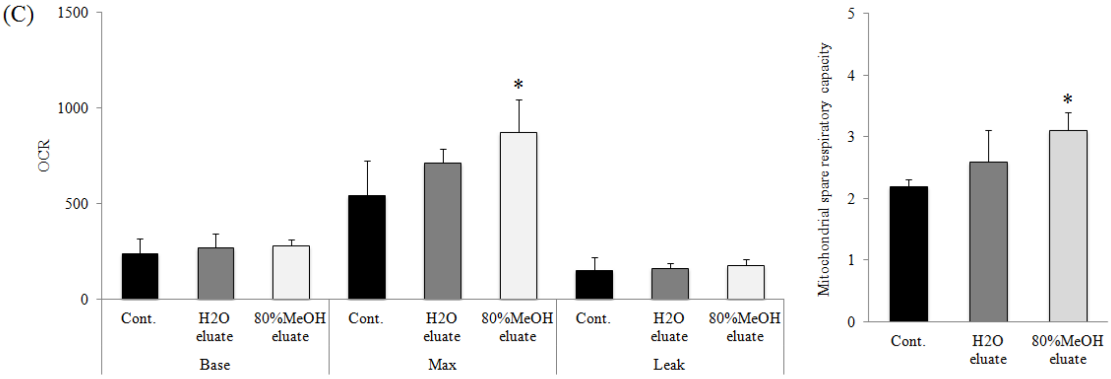

Supplemental Figure 1. Analysis of Amla functional components. Amla extract (50 g) was separated by Diaion HP-20 column chromatography (Mitsubishi Chemical Corporation, Tokyo, Japan) using H_2O as an eluant, followed by elution with 80% MeOH. Each eluate was lyophilized to retrieve the H_2O eluate (30.2 g, 60.4%) and the 80% MeOH eluate (14.6 g, 29.2%). These products were then applied to component and mitochondrial function analyses as described in Section 2 of the manuscript. (A) Schematic figure describing Amla fractionation. (B) Component analysis of the two Amla fractions. (C) The effects of the Amla fractions on mitochondrial function in C2C12 myotubes. The fractions were treated in equivalent doses to Amla extract (200 µg/mL). *$p < 0.05$, as compared with control (n=5 or 6).
